# Ovarian cancer

**DOI:** 10.1054/bjoc.2000.1758

**Published:** 2001-05

**Authors:** P Kerbrat, C Lhommé, B Fervers, J P Guastalla, L Thomas, N Tournemaine, J P Basuyau, C Cohen-Solal, P Duvillard, T Bachelot, I Ray, E Voog, J Dauplat

**Affiliations:** Centre Eugène Marquis, Rennes; Institut Gustave Roussy, Villejuif; Centre Léon Bérard, Lyon; Cancer Research Campaign, Paris; Institut Bergonié, Bordeaux; Centre Catherine de Sienne, Nantes; Centre Henri Becquerel, Rouen; Centre René Huguenin, Saint-Cloud; Centre Jean Perrin, Clermont-Ferrand, France


British Journal of Cancer (2001) 84(Supplement 2), 18–23
© 2001 FNCLCC
doi: 10.1054/ bjoc.2001.1758, available online at http://www.idealibrary.com on IDEAL®

### Ovarian cancer

P Kerbrat[1], C Lhommé[2], B Fervers[3,4], JP Guastalla[3], L Thomas[5], N Tournemaine[6], JP Basuyau[7], C Cohen-Solal[8], P Duvillard[2], T Bachelot[3], I Ray[3], E Voog[3] and J Dauplat[9]

[1]Centre Eugène Marquis, Rennes; [2]Institut Gustave Roussy, Villejuif; [3]Centre Léon Bérard, Lyon; [4]FNCLCC, Paris; [5]Institut Bergonié, Bordeaux; [6]Centre Catherine de Sienne, Nantes; [7]Centre Henri Becquerel, Rouen; [8]Centre René Huguenin, Saint-Cloud; [9]Centre Jean Perrin, Clermont-Ferrand, France

Epithelial ovarian cancer is responsible for 5.8% of cancer deaths. The mortality rate increases with age and two-thirds of the deaths occur after the age of 65. Seventy-five percent of these tumours are at an advanced stage (III or IV) at the time of diagnosis. The overall 5-year survival is about 30%.

This SOR covers only the initial management of epithelial ovarian cancer. It does not consider the management of recurrent disease. Similarly it does not discuss tumours of low malignant potential (borderline tumours), germ cell tumours or the management of women with a genetic predisposition to ovarian adenocarcinoma.

The SOR document for the management of epithelial ovarian cancer was originally published in March 1998. The recommendations were reviewed and updated by the ovarian working group in April 1999. The next update is planned for 2001.

### SCREENING

For pre- or postmenopausal women without a family history of ovarian cancer, routine population or individual screening programmes by ultrasound and/or CA125 assay is not indicated (standard). There is no risk-factor sufficiently powerful to identify a population large enough for screening to have any benefit in public health terms. Patients with a family history of ovarian cancer can be offered a genetic cancer consultation (option). In women with a genetically defined risk of ovarian cancer, or in familial forms of ovarian cancer, screening is not recommended outside a formal trial. In women with a definite genetic predisposition to cancer of the ovary, or in familial forms, it is recommended to set up targeted screening programmes, but only within a properly evaluated programme.

### DIAGNOSIS

A pelvic mass detected clinically or an ovarian mass seen on ultrasound raises the possibility of ovarian cancer. An ovarian cancer has certain characteristic features on suprapubic and transvaginal pelvic ultrasonography (level of evidence B), but the definitive diagnosis of malignancy can only be made histologically.

The ultrasound report must be complete and unambiguous. It must document:

- the technique used
- the phase of the menstrual cycle in premenopausal women
- the size and topography of the lesion(s)
- whether it is uni- or bilateral
- whether it is entirely fluid filled, mixed, predominantly fluid or predominantly solid
- whether any solid component is homogeneous or heterogeneous.

In the case of a cystic or predominantly cystic lesion, the report must describe the thickness of the walls, the presence of septae (single or multiple), the presence of papillae or endocystic or exocystic vegetations and whether there are any associated signs (e.g. fluid in the pouch of Douglas, ascites, lymphadenopathy, hydronephrosis).

Transvaginal colour doppler ultrasonography is not indicated at diagnosis outside a controlled study. Its reliability in differentiating benign from malignant ovarian tumours has yet to be proven (level of evidence B). No other methods of imaging are indicated routinely during initial evaluation. In the case of diagnostic difficulty, ultrasonography may be complimented by CT scanning or MRI (options). MRI gives the best anatomical detail when evaluating large masses of uncertain origin (uterus or ovary). Similarly, MRI may be of use in trying to distinguish tumour from certain benign lesions (e.g. endometriosis, or some mature teratomas). The information gained may help decide what kind of surgery should be undertaken (i.e. laparoscopy or laparotomy) and whether this should be carried out electively in a cancer centre.

### Evaluation of cysts – fine-needle aspiration for cytology

Fine-needle aspiration by the transabdominal or transvaginal route for the cytological examination of all ovarian masses (solid or mixed) is contra-indicated (standard). The management of purely fluid cysts is different in premenopausal and post-menopausal women.

**i)** Post-menopausal women. Transvaginal aspiration of a purely fluid, echogenic, thin-walled cyst of more than 5 cm diameter without endocystic vegetations is contra-indicated (standard). These must be followed, either by ultrasonography, or excised by a gynaecological surgeon (standard). There is no consensus as to the management of clear cysts of less than 5 cm in post-menopausal women.

**ii)** Pre-menopausal women. There is no consensus as to the ideal management. Fine-needle aspiration of purely fluid cysts with echogenic contents and without endocystic vegetations is highly controversial. If there is a high probability of the cyst being benign and functional many gynaecologists will choose to aspirate the cyst via the transvaginal route under ultrasound control. There are reservations about this practice because of the difficulty in interpreting diagnostic ultrasound in all patients, the results being operator-dependent. The risk of a non-informative cytological examination following aspiration is in the order of 25% and the risk of recurrence is about 20%.

### STAGING

Preoperative imaging is not sufficient for precise staging. Except for cases presenting with stage IV disease (e.g. with a pleural effusion or supraclavicular nodes), surgical exploration and histopathological examination are necessary for confirmation of the histological diagnosis and for definitive staging (standard, level of evidence B). CT scanning is not a useful preoperative staging tool (standard, level of evidence B). MRI is only useful in certain cases, for example, when surgical management will be altered if there is involvement of the rectosigmoid, bladder or pelvic wall (level of evidence B).

### POSTOPERATIVE IMAGING

If all macroscopic disease has been removed at initial surgery, a postoperative CT scan should not be done routinely. In the case of extensive complete surgery resulting in major anatomical disruption, a postoperative abdomino-pelvic CT scan can facilitate subsequent on-treatment evaluation (option). If initial surgery has been incomplete, a baseline CT scan should be taken 3 weeks to 1 month after surgery, or immediately before the start of chemotherapy, in order to follow subsequent response to treatment. Similarly, a baseline abdomino-pelvic CT scan is necessary if initial surgery is contra-indicated (e.g. if the patient is of poor performance status) and when chemotherapy is to be given as primary treatment (level of evidence B).

### TUMOUR MARKERS

A serum CA125 assay before surgery and before the start of chemotherapy is standard. Other markers (ACE, CA19.9) should only be measured if the serum CA125 is not elevated at diagnosis, notably in the case of mucinous or endometrioid tumours. In young women, measurement of alpha-foetoprotein and beta-HCG is recommended to exclude a germ cell tumour.

The measurement of CA125 levels during chemotherapy is standard. An assay before each of six courses and a month after the last course is recommended.

### EXTRAOVARIAN PERITONEAL CARCINOMA

Malignant ovarian cancers presenting as peritoneal carcinomatosis must be treated surgically without delay in a specialist cancer setting (standard, level of evidence B). Assessment includes an abdomino-pelvic ultrasound and the aspiration of ascitic fluid for cytological analysis (standard), which may be followed by a CT scan of the abdomen and pelvis (option). If an ovarian origin is likely, there is no point in carrying out further investigations. These are only appropriate if an alternative origin is suspected. For patients whose performance status is too poor for surgery and who would benefit from primary chemotherapy, a CT scan of the abdomen and pelvis is undertaken as a base-line for follow-up on treatment.

### HISTOLOGICAL DIAGNOSIS

Good quality, adequately sized representative samples from the ovarian tumour(s) must be examined (standard). Peritoneal biopsies are not sufficient; they do not provide proof of ovarian origin, nor the degree of malignancy (standard).

A meticulous macroscopic examination taking into account the clinical details must precede histological analysis. A medical summary should accompany the surgically excised tissue samples. The excised tissue, intact and unopened, must be transported as rapidly as possible to the laboratory (standard). The choice of fixative is very important and must be appropriate for all analytical tests that might be used (standard).

The analysis should include:

- the weight and measurement of all samples
- a detailed macroscopic description of all excised pieces
- multiple well directed samples from all ovarian lesion(s) (one per centimetre along the greatest diameter), any extra-ovarian spread and routine biopsies taken as part of surgical staging (standard).

The routine freezing of representative samples is recommended. The intraoperative examination (frozen section) of ovarian lesion(s) can guide surgery. Interpretation may be difficult for epithelial tumours of the ovary and requires a good knowledge of the clinical details and close collaboration with the surgical team. The main limitation of a frozen section is the distinction between very well differentiated adenocarcinomas and borderline tumours.

Immuno-markers can be used to classify a poorly differentiated tumour as an epithelial tumour, but unfortunately, none of the commercially available immuno-marker kits are specific for these tumour types.

### PATHOLOGICAL REPORTING

Macroscopic features must be described precisely and include all the samples received. Similarly, the histological description must be precise and provide all the information necessary to make a diagnosis of malignancy. It must also contain all factors necessary for staging and prognosis: histological type, degree of differentiation and description of any extra-ovarian spread. The conclusion

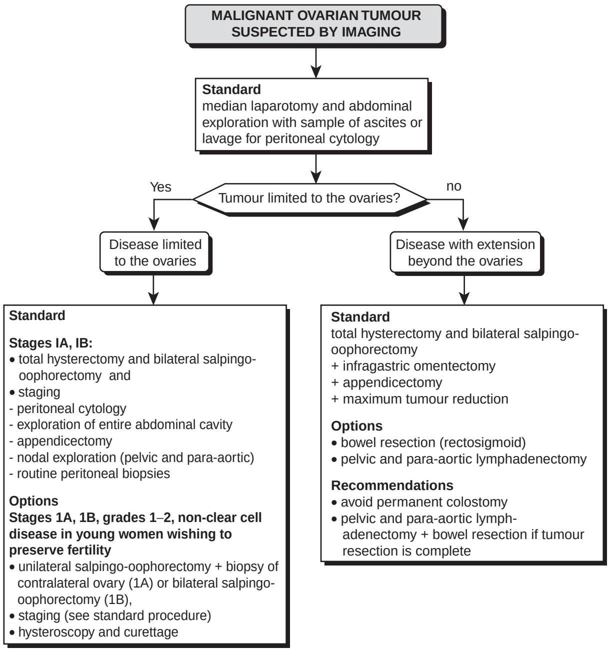

**Figure 1** Surgery for ovarian cancer

© 2001 FNCLCC

of the report must be clear and summarize both the macroscopic and the histological findings. It must specify the side, the presence or absence of exophytic vegetations and/or of a ruptured capsule, the histological type, degree of differentiation and whether there is any extension to pelvic or abdominal organs or to lymph nodes. In cases of extensive surgery, it must also describe the quality of the excision margins. Finally, it must include the results of the cytological examination of ascitic fluid or peritoneal lavage.

### CLASSIFICATION

The standard classification of ovarian tumours is that of the International Federation of Gynaecologists and Obstetricians (FIGO) (standard). It is a pathological classification determined after initial surgery.

### PROGNOSTIC FACTORS

The independent prognostic factors that determine treatment modalities are tumour extension (FIGO stage), the volume of residual disease after initial surgery, the histological type, the degree of differentiation (especially for the early stage disease), age and performance status.

### THERAPEUTIC STRATEGY

### Surgery for cancer of the ovary

Surgery for ovarian cancer should only be carried out by those with extensive experience in oncology, gynaecology, general surgery and endoscopic surgery. It should only be undertaken by teams able to offer a multidisciplinary approach for the management of these patients.

Surgery for early stage disease (Figure 1) has two main aims: adequate staging and the conservation of fertility (if this is possible and the patient's wish). Surgery for advanced disease is based on the general principle of maximum tumour reduction with the awareness that ultraradical surgery is a controversial issue. Surgery for re-evaluation (second-look surgery) has a role in clinical trials.

The operative report must include a precise and detailed description of the operative findings and of the surgery undertaken. If a patient presents to a specialist centre following surgery that is considered to be inadequate, a further staging operation should be undertaken. Similarly, staging surgery should be undertaken as soon as possible if a diagnosis of ovarian adenocarcinoma has been made following minor surgery (e.g. laparoscopy). A restaging operation after laparoscopy must include the resection of trochar tracks.

#### *Surgical treatment of early stage disease limited to the ovaries (stages IA, IB)*

The standard surgical treatment for tumour limited to the ovaries in post-menopausal women and women not wanting children is total hysterectomy and bilateral salpingo-oophorectomy (TAH, BSO) by para-median incision. Complete staging consists of collection of ascitic fluid or peritoneal lavage for cytology, exploration of the entire abdominal cavity, infracolic omentectomy and appendicectomy, assessment of pelvic and para-aortic nodes and routine peritoneal biopsies. There is no consensus as to the best means of assessing retroperitoneal nodes. The therapeutic value of lymphadenectomy has not been demonstrated, but it may be the only way of excluding nodal metastases in early stage disease and confirming stage I disease.

In women with stages IA, IB, grades 1 or 2, non-clear cell disease wishing to maintain their fertility, conservative surgery is possible (option). If the tumour is unilateral, surgery consists of the complete staging procedure plus unilateral oophorectomy and examination of the contralateral ovary. Bilateral oophorectomy is mandatory for bilateral tumours. An assessment of the uterine cavity by hysteroscopy and curettage is mandatory in all cases. Total hysterectomy with bilateral oophorectomy along with the full staging procedure is recommended in women in whom fertility is no longer desired.

#### *Surgical treatment of disease with extraovarian spread (stages IC, II, III, IV)*

Standard treatment for patients presenting with advanced stage disease consists of BSO by para-median incision with complete excision of the lumbar-ovarian vessels, a total hysterectomy with vaginal closure, a complete infragastric omentectomy and an appendicectomy. The volume of tumour left in place after initial surgery is of prognostic value. Patients without residual disease (complete excision) or minimal residue (optimal excision) have a better chance of a prolonged survival. Patients who have significant residual tumour (sub-optimal excision) or who have only had a biopsy, have a very poor prognosis.

If the operation has resulted in complete or optimal tumour resection, pelvic and para-aortic lymphadenectomy can be justified on several grounds: some experts consider this to be a therapeutic measure, metastatic nodes are markers of distant metastases, and involved nodes can persist after chemotherapy.

If a standard operation does not result in a complete or optimal resection, additional interventions can be undertaken, including excision of the entire genital tract, bowel resection and excision of peritoneal metastases. Pelvic and para-aortic lymphadenectomy can be undertaken if the abdominal tumour resection has been complete or optimal and there is nodal enlargement.

If a standard operation does not result in a complete or optimal resection, chemotherapy can be started immediately and two or three courses given before a second attempt at interval debulking surgery (option). If the standard operation is likely to be difficult (e.g. if the patient is in poor general health or has a fixed pelvis and in some cases of stage IV disease), a limited exploration (by laparotomy or laparoscopy) can be undertaken for precise staging and for ovarian biopsies. The patient may then proceed rapidly to two or three courses of chemotherapy before interval debulking surgery is attempted (option).

If a patient is referred to a specialist centre after a sub-optimal resection, either a further operation can be undertaken immediately in an attempt to complete tumour resection (option), or two or three courses of chemotherapy can be given before interval debulking surgery (option).

Tumour resection must be as complete as possible. Ultra-radical surgery must only be considered if a prolonged postoperative course would not delay the start of chemotherapy.

The preoperative bowel preparation should be identical to that used for bowel surgery. If a recto-sigmoid resection is necessary, a low colorectal anastomosis should be fashioned if at all possible. A reversible colostomy is a possible option. A permanent colostomy will have a deleterious effect on patient quality of life. The operation report must include a detailed description of all lesions prior

*© 2001 FNCLCC*

to excision, a precise description of the surgery undertaken and a precise description of the size and the localization of residual tumour left in place.

### *Surgery for re-evaluation and secondary tumour debulking*

Surgery for re-evaluation or 'second look' surgery is practiced after initial standard surgery and first-line chemotherapy in a patient in complete remission as judged clinically, by imaging and by tumour markers. It is not recommended routinely as it has not been shown to benefit patients (standard, level of evidence C). It can be considered if it is likely to change subsequent therapy (option) or as part of a therapeutic trial. It can be done by laparotomy or by laparoscopy (option) if the operator is trained in the technique.

### *Surgical management if initial surgery has not been done according to standard procedures*

This applies particularly if lymphadenectomy has been omitted. A second operation can be undertaken, as studies have shown that metastatic nodes can persist despite chemotherapy. Secondary cytoreduction surgery for recurrence, or after completion of first-line treatment, is controversial and has no proven benefit (level of evidence C). It should only be considered if there are favourable features such as a single mass or several masses which are likely to be completely excised and in cases of late relapse. It should not be considered in patients progressing on chemotherapy or in those with disseminated peritoneal disease.

### Adjuvant treatment

### *Adjuvant treatment for stage I and IIA disease (following optimal staging and with histological grading)*

There is no indication for adjuvant treatment for stage IA GI disease (standard) (Figure 2). There is no standard for stage IA, G2–3 or clear cell tumours, IB, IC or IIA disease. The options are: no additional treatment, chemotherapy based on platinum and external beam abdomino-pelvic irradiation.

For stage I disease, there is insufficient evidence to show that adjuvant treatment (chemotherapy or abdomino-pelvic external radiotherapy) improves overall survival (level of evidence C) but there is a benefit from cisplatin-based chemotherapy in terms of survival without recurrence, as compared to follow-up alone (level of evidence C).

For stage IA G2–3 or clear cell tumours, IB, IC and IIA disease, external abdomino-pelvic radiotherapy, chemotherapy or no additional treatment are options (level of evidence C). Additional treatment is recommended for stage IC, IIA disease and for all stages with G3 or clear cell tumours.

The available data is not sufficient to differentiate the efficacy of chemotherapy and external radiotherapy (level of evidence C), but the chemotherapy regimens used in the comparative trials were sub-optimal as compared to the chemotherapy currently used for advanced disease. Toxicity is more severe (with a higher treatment 'drop-out' rate) with external radiotherapy as compared to chemotherapy (level of evidence C).

External irradiation should include the entire abdomino-pelvic cavity (abdomino-pelvic irradiation) (standard). Irradiation of the pelvis alone is not recommended (standard, level of evidence B). Abdomino-pelvic irradiation requires the use of a linear accelerator, with an open field technique using two antero-posterior or four orthogonal beams with kidney shields at 15 Gy and liver shields at 20 Gy. The exact status of the abdominal and pelvic cavities must be taken into account before undertaking abdomino-pelvic radiotherapy; there must be no macroscopic residual disease. Toxicity is increased in patients who have undergone multiple previous operations and in those receiving pelvic boosts (level of evidence C).

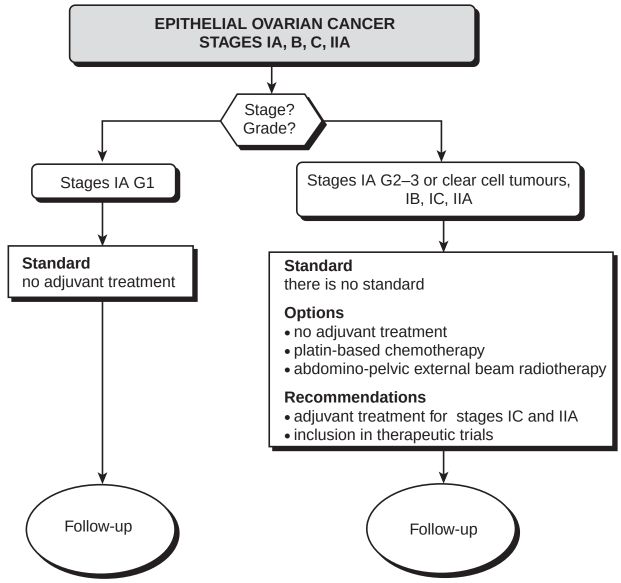

**Figure 2** Adjuvant treatment of stages IA, IB, IC, IIA epithelial ovarian cancer

Intraperitoneal brachytherapy is not recommended (level of evidence B).

Patients should be considered for entry into therapeutic trials.

### *Adjuvant treatment for stage IIB, IIC and III disease (without residual disease and following optimal staging)*

There is no standard for stage IIB, IIC and III tumours without disease (Figure 3). The options are platinum-based chemotherapy and external abdomino-pelvic radiotherapy.

For stage IIB, IIC and III tumours without residual disease, adjuvant treatment is recommended. The available data is not sufficient to differentiate the relative efficacy of chemotherapy and external radiotherapy (level of evidence C), but the chemotherapy used in comparative trials has been sub-optimal as compared to current regimens. The nature of the toxicity of the two treatments is different (level of evidence B).

External radiotherapy must include the entire abdomino-pelvic cavity (abdomino-pelvic radiotherapy) (standard). Toxicity is increased following repeated surgery and with pelvic boosting (level of evidence C).

Intra-peritoneal brachytherapy is not recommended (level of evidence B).

Patients should be included in therapeutic trials.

### *Additional treatment in advanced disease (IIB, IIC, III with residual disease and IV)*

The standard treatment for advanced disease is combination chemotherapy with an intravenous platin (Figure 4) at a dose-intensity of 25 mg $m^{-2}$ $week^{-1}$ of cisplatin (75 mg $m^{-2}$ over 3 weeks) or the equivalent dose of carboplatin, for at least six courses (level of evidence C).

© 2001 FNCLCC

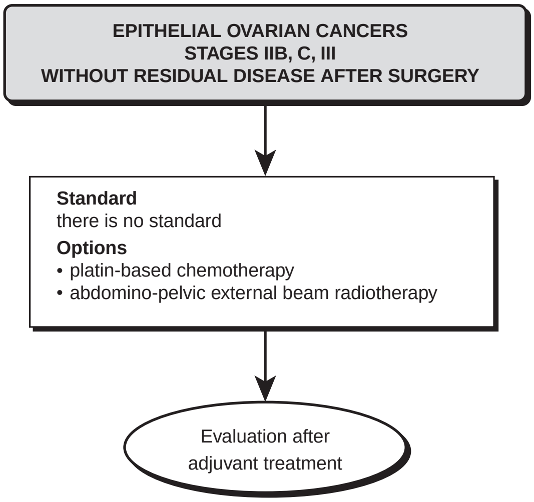

**Figure 3** Adjuvant treatment of stages IIB, IIC, III epithelial ovarian cancer, without residual disease after surgery

The options are:

- an intravenous platin combined with intravenous paclitaxel
- an intravenous platin combined with intravenous cyclophosphamide and/or doxorubicin
- intraperitoneal cisplatin combined with intravenous cyclophosphamide.

Intravenous carboplatin has equivalent efficacy to cisplatin (level of evidence A) when given at an equivalent dose of 4:1. The non-haematological toxicity of carboplatin is less than that of cisplatin, but the haematological toxicity is greater (level of evidence B). The optimal number of cycles of chemotherapy is unknown. The available data does not support a benefit in terms of overall survival in giving more than six courses (level of evidence C).

There is a significant survival benefit with the addition of doxorubicin to the cyclophosphamide, cisplatin combination (level of evidence A). This benefit is seen even at doses that are sub-optimal compared to current standards. It is not clear if the benefit is a result of an increase in overall dose intensity or the addition of

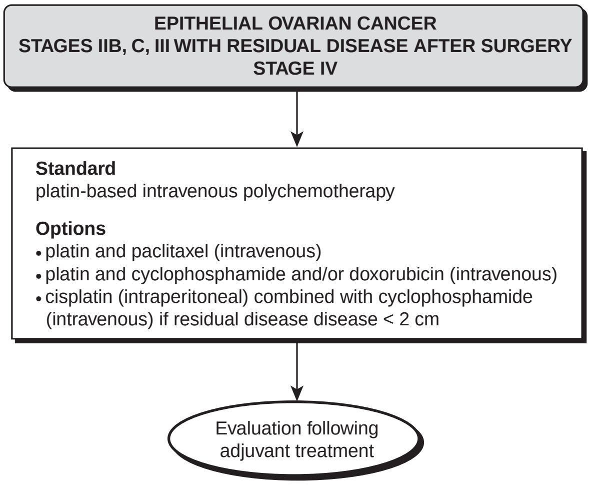

**Figure 4** Adjuvant treatment of stages IIB, IIC, III epithelial ovarian cancer with residual disease after surgery

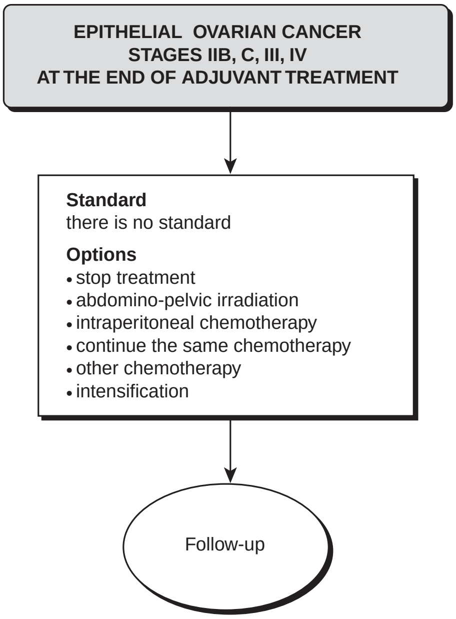

**Figure 5** Management of stages IIB, IIC, III, IV epithelial ovarian cancer at the end/after adjuvant treatment doxorubicin per se. The additional toxicity related to the doxorubicin was not evaluated in the meta-analysis.

In stage III disease with residual disease post-surgery of ≥ 1 cm and for stage IV, the combination of cisplatin-paclitaxel leads to an increase in overall survival compared to the cisplatin-cyclophosphamide combination (level of evidence B). This is also true for residual disease ≤ 1 cm (level of evidence B). When paclitaxel has not been used as first-line chemotherapy, it is recommended that it be used for recurrent disease.

In the only study in which intraperitoneal cisplatin has been combined with intravenous cyclophosphamide and compared to intravenous cisplatin in stage III disease, an increase in overall survival was seen in the intraperitoneal treatment arm for cases with residual disease post initial surgery of < 2 cms (level of evidence B).

External beam abdomino-pelvic radiotherapy and intraperitoneal brachytherapy have no place in first-line treatment of advanced disease where there is residual disease after surgery.

The use of immunotherapy or hormone therapy is not recommended as first line treatment in advanced disease (level of evidence C).

### *Consolidation treatment*

There is no standard treatment after second-look surgery (Figure 5). In the absence of macroscopic disease, there is no difference in overall survival between chemotherapy and external abdomino-pelvic irradiation when used as consolidation treatment (level of evidence C). The toxicity and toxicity-related ‘drop out’ rates are higher for external radiotherapy compared to chemotherapy (level of evidence C).

There is little evidence to support any of the following options: no treatment, more chemotherapy of the same type, intraperitoneal chemotherapy, abdomino-pelvic irradiation or high-dose chemotherapy. This is irrespective of whether there has been a complete clinical or histological response, minimal macroscopic or microscopic residual disease.

 *© 2001 FNCLCC*

The effect of changing to another chemotherapy protocol after six cycles of chemotherapy has not been evaluated.

When macroscopic residual disease is found at second-look surgery, abdomino-pelvic radiotherapy is not recommended (standard, level of evidence C).

Alpha-interferon combined with intraperitoneal carboplatin after platin-based chemotherapy and second look surgery does not improve survival (level of evidence C).

It is recommended that patients be included in therapeutic trials.

### HORMONE REPLACEMENT THERAPY

There is no standard. Hormone replacement therapy (HRT) after the menopause has not been shown to be associated with the development of ovarian adenocarcinomas in epidemiological studies (level of evidence B). There is no epidemiological, biological or clinical data to preclude the use of HRT in patients previously treated for cancer of the ovary in the absence of other contra-indications to HRT. There is no evidence that HRT is associated with the recurrence of ovarian cancer after treatment. Breast surveillance is recommended in these patients because of the possible association of ovarian and breast cancers. There is no consensus as to the combined use of progestogens and oestrogens following hysterectomy.

### POST-TREATMENT ASSESSMENT

Assessment at the end of chemotherapy includes clinical examination, measurement of tumour markers (in particular CA125) and a CT scan of the abdomen and pelvis (level of evidence B) to identify any residual mass (standard). Abdominal ultrasound should be used in addition to CT scanning to monitor parenchymal hepatic or splenic metastases (level of evidence B) (option).

If there is doubt as to the persistence of disease in the region of the right hemi-diaphragm, MRI should be used (level of evidence B) (option). A normal CA125 level after six courses of chemotherapy does not confirm a histological complete response, whereas an elevated serum level at this time confirms the absence of a complete response. Because of the variability in results with different methods, each patient should be followed using the same test method, preferably carried out at the same laboratory.

### FOLLOW-UP

The follow-up of patients without clinical signs is based on clinical examination (standard). In the absence of curative treatment for relapsed disease, there is no consensus as to the necessity and frequency of CA125 assays (option) during follow-up. Surveillance by CT and ultrasonography are only indicated if tumour markers have not been reliable. Surveillance by MRI is not recommended (level of evidence B). There are no data to define the frequency of surveillance.

If there is an increase in a previously normal CA125 level, the assay should be repeated after 2–3 weeks to confirm the increase and to calculate the time of doubling or the rate of progression. If a relapse is suspected in the presence of clinical signs and/or a confirmed elevation of tumour markers, the first investigation to undertake is a CT scan of the abdomen and pelvis. An abdomino-pelvic ultrasound need only be done if the CT is negative. Immuno-scintigraphy is only indicated if both the CT and ultrasound are negative (level of evidence B).

Exceptions might apply for patients in clinical trials.

### INTERNAL REVIEWERS

C Bailly (Centre Léon Bérard, Lyon), JM Bidart (Institut Gustave Roussy, Villejuif), YJ Bignon (Centre Jean Perrin, Clermont), V Cabaret (Centre Oscar Lambret, Lille), B Castelain (Centre Oscar Lambret, Lille), A Chevalier (Centre Oscar Lambret, Lille), H Crouet (Centre Francois Baclesse, Caen), A Delobelle (Centre Oscar Lambret, Lille), R Delva (Centre Paul Papin, Angers), P Dessogne (Centre Henri Becquerel, Rouen), JP Droz (Centre Léon Bérard, Lyon), JC Durand (Institut Curie, Paris), F Eisinger (Institut Paoli Calmettes, Marseille), E Gougeon (Centre Oscar Lambret, Lille), A Goupil (Centre Renée Huguenin, Saint-Cloud), G Houvenaeghel (Institut Paoli Calmettes, Marseille), E Lartigau (Institut Gustave Roussy, Villejuif), S Lasry (Centre René Huguenin, Saint-Cloud), E Leblanc (Centre Oscar Lambret, Lille), D Lebrun-Jezekova (Institut Jean Godinot, Reims), P Liégeois (Centre Francois Baclesse, Caen), G Lorimier (Centre Paul Papin, Angers), I Martel (Centre Léon Bérard, Lyon), P Martel (Centre Caludius Regaud, Toulouse), F Mayer (Centre Georges-François Leclerc, Dijon), G Michel (Institut Gustave Roussy, Villejuif), E Netter (Centre Alexis Vautrin, Nancy), TD Nguyen (Institut Jean Godinot, Reims), A Pavret de la Rochefordière (Institut Curie, Paris), M Peffault de Latour (Centre Jean Perrin, Clermont-Ferrand), T Petit (Centre Paul Strauss, Strasbourg), MF, Pichon (Centre René Huguenin, Saint-Cloud), V Servent (Centre Oscar Lambret, Lille), F Spyratos (Centre René Huguenin, Saint-Cloud), E Stöckle (Institut Bergonié, Bordeaux), S Taieb (Centre Oscar Lambret, Lille), P Troufléau (Centre Alexis Vautrin, Nancy), M Tubiana (Centre René Huguenin, Saint-Cloud), P Vennin (Centre Oscar Lambret, Lille), JL Verhaeghe (Centre Alexis Vautrin, Nancy), MO Vilain (Centre Oscar Lambret, Lille) and B Weber (Centre Alexis Vautrin, Nancy).

### EXTERNAL REVIEWERS

G Brun (GH Saint-André Jean Abadie, Bordeaux), L Frappart (Hôpital Edouard Herriot, Lyon), X Froger (Centre Hospitalier, Chambéry), MC Gouttebel (Centre Hospitalier, Roanne), D Khayat (Hôpital de la Pitié Salpétrière, Paris), J Lansac (Hôpital Bretonneau, Tours), JP Le Bourgeois (Hôpital Henri Mondor, Créteil), O Le Floch (Hôpital Bretonneau, Tours), D Querleu (CHU-Pavillon Paul Gellé, Roubaix), JC Rymer (Hôpital Henri Mondor, Créteil), M Debeard (Lille), M Bethouart (Roubaix), B Chauvet (Clinique Sainte-Catherine, Avignon), T Facchini (Clinique Courlancy, Reims), D Fric (Clinique du Mail, Grenoble), H Lauche (Clinique Clémentville, Montpellier), N Rouverand (Clinique de la Louvière, Lille), P Vandenbussche (Lille), J Vignoud (Clinique Clémentville, Montpellier) and R Villet (Hôpital des Diaconnesses, Paris).

© 2001 FNCLCC

